# The Icarian flight of antibody-drug conjugates: target selection amidst complexity and tackling adverse impacts

**DOI:** 10.1093/procel/pwaf002

**Published:** 2025-02-25

**Authors:** Han Liu, Hongye Zeng, Xiaojing Qin, Wenjing Ning, Lin Xu, Shiting Yang, Xue Liu, Wenxin Luo, Ningshao Xia

**Affiliations:** State Key Laboratory of Vaccines for Infectious Diseases, Xiang An Biomedicine Laboratory, School of Public Health, Xiamen University, Xiamen 361102, China; National Institute of Diagnostics and Vaccine Development in Infectious Diseases, State Key Laboratory of Molecular Vaccinology and Molecular Diagnostics, National Innovation Platform for Industry-Education Integration in Vaccine Research, the Research Unit of Frontier Technology of Structural Vaccinology of Chinese Academy of Medical Sciences, Xiamen University, Xiamen 361102, China; State Key Laboratory of Vaccines for Infectious Diseases, Xiang An Biomedicine Laboratory, School of Public Health, Xiamen University, Xiamen 361102, China; National Institute of Diagnostics and Vaccine Development in Infectious Diseases, State Key Laboratory of Molecular Vaccinology and Molecular Diagnostics, National Innovation Platform for Industry-Education Integration in Vaccine Research, the Research Unit of Frontier Technology of Structural Vaccinology of Chinese Academy of Medical Sciences, Xiamen University, Xiamen 361102, China; State Key Laboratory of Vaccines for Infectious Diseases, Xiang An Biomedicine Laboratory, School of Public Health, Xiamen University, Xiamen 361102, China; National Institute of Diagnostics and Vaccine Development in Infectious Diseases, State Key Laboratory of Molecular Vaccinology and Molecular Diagnostics, National Innovation Platform for Industry-Education Integration in Vaccine Research, the Research Unit of Frontier Technology of Structural Vaccinology of Chinese Academy of Medical Sciences, Xiamen University, Xiamen 361102, China; State Key Laboratory of Vaccines for Infectious Diseases, Xiang An Biomedicine Laboratory, School of Public Health, Xiamen University, Xiamen 361102, China; National Institute of Diagnostics and Vaccine Development in Infectious Diseases, State Key Laboratory of Molecular Vaccinology and Molecular Diagnostics, National Innovation Platform for Industry-Education Integration in Vaccine Research, the Research Unit of Frontier Technology of Structural Vaccinology of Chinese Academy of Medical Sciences, Xiamen University, Xiamen 361102, China; State Key Laboratory of Vaccines for Infectious Diseases, Xiang An Biomedicine Laboratory, School of Public Health, Xiamen University, Xiamen 361102, China; National Institute of Diagnostics and Vaccine Development in Infectious Diseases, State Key Laboratory of Molecular Vaccinology and Molecular Diagnostics, National Innovation Platform for Industry-Education Integration in Vaccine Research, the Research Unit of Frontier Technology of Structural Vaccinology of Chinese Academy of Medical Sciences, Xiamen University, Xiamen 361102, China; State Key Laboratory of Vaccines for Infectious Diseases, Xiang An Biomedicine Laboratory, School of Public Health, Xiamen University, Xiamen 361102, China; National Institute of Diagnostics and Vaccine Development in Infectious Diseases, State Key Laboratory of Molecular Vaccinology and Molecular Diagnostics, National Innovation Platform for Industry-Education Integration in Vaccine Research, the Research Unit of Frontier Technology of Structural Vaccinology of Chinese Academy of Medical Sciences, Xiamen University, Xiamen 361102, China; State Key Laboratory of Vaccines for Infectious Diseases, Xiang An Biomedicine Laboratory, School of Public Health, Xiamen University, Xiamen 361102, China; National Institute of Diagnostics and Vaccine Development in Infectious Diseases, State Key Laboratory of Molecular Vaccinology and Molecular Diagnostics, National Innovation Platform for Industry-Education Integration in Vaccine Research, the Research Unit of Frontier Technology of Structural Vaccinology of Chinese Academy of Medical Sciences, Xiamen University, Xiamen 361102, China; State Key Laboratory of Vaccines for Infectious Diseases, Xiang An Biomedicine Laboratory, School of Public Health, Xiamen University, Xiamen 361102, China; National Institute of Diagnostics and Vaccine Development in Infectious Diseases, State Key Laboratory of Molecular Vaccinology and Molecular Diagnostics, National Innovation Platform for Industry-Education Integration in Vaccine Research, the Research Unit of Frontier Technology of Structural Vaccinology of Chinese Academy of Medical Sciences, Xiamen University, Xiamen 361102, China; State Key Laboratory of Vaccines for Infectious Diseases, Xiang An Biomedicine Laboratory, School of Public Health, Xiamen University, Xiamen 361102, China; National Institute of Diagnostics and Vaccine Development in Infectious Diseases, State Key Laboratory of Molecular Vaccinology and Molecular Diagnostics, National Innovation Platform for Industry-Education Integration in Vaccine Research, the Research Unit of Frontier Technology of Structural Vaccinology of Chinese Academy of Medical Sciences, Xiamen University, Xiamen 361102, China

**Keywords:** antibody-drug conjugates, target selection, normal and tumor tissue, off-target toxicity, response strategies

## Abstract

Antibody-drug conjugates (ADCs) represent a promising class of targeted cancer therapeutics that combine the specificity of monoclonal antibodies with the potency of cytotoxic payloads. Despite their therapeutic potential, the use of ADCs faces significant challenges, including off/on-target toxicity and resistance development. This review examines the current landscape of ADC development, focusing on the critical aspects of target selection and antibody engineering. We discuss strategies to increase ADC efficacy and safety, including multitarget approaches, pH-dependent antibodies, and masked peptide technologies. The importance of comprehensive antigen expression profiling in both tumor and normal tissues is emphasized, highlighting the role of advanced technologies, such as single-cell sequencing and artificial intelligence, in optimizing target selection. Furthermore, we explore combination therapies and innovations in linker‒payload chemistry, which may provide approaches for expanding the therapeutic window of ADCs. These advances pave the way for the development of more precise and effective cancer treatments, potentially extending ADC applications beyond oncology.

## Background

There have been three significant revolutions in the landscape of oncological pharmacotherapy. In 1948, the era of chemotherapeutic drugs for cancer treatment began (Miller, 2006). In the 1990s, there was a second revolution in the oncology field, which was characterized by the emergence and development of small molecule inhibitors as targeted therapeutic agents (Saikia, 2018). In the late 1990s, the third revolution began with the advent of tumor immunotherapy. In 2011, the successful clinical trial and subsequent market launch of ipilimumab (Yervoy) was a seminal moment in the immunotherapy era, signalling the development of bona fide cancer immunotherapeutic agents (Mellman et al., 2011). Although monoclonal antibody therapy is effective in certain cases, several limitations of the use of this approach in tumor treatment, including insufficient specificity, development of resistance, and inhibitory effects of the tumor microenvironment, have been observed over time. These challenges highlight the need for more diversified therapeutic options. To overcome the aforementioned limitations, the scientific community has developed an innovative therapeutic approach known as antibody‒drug conjugates (ADCs).

**Figure 1.**
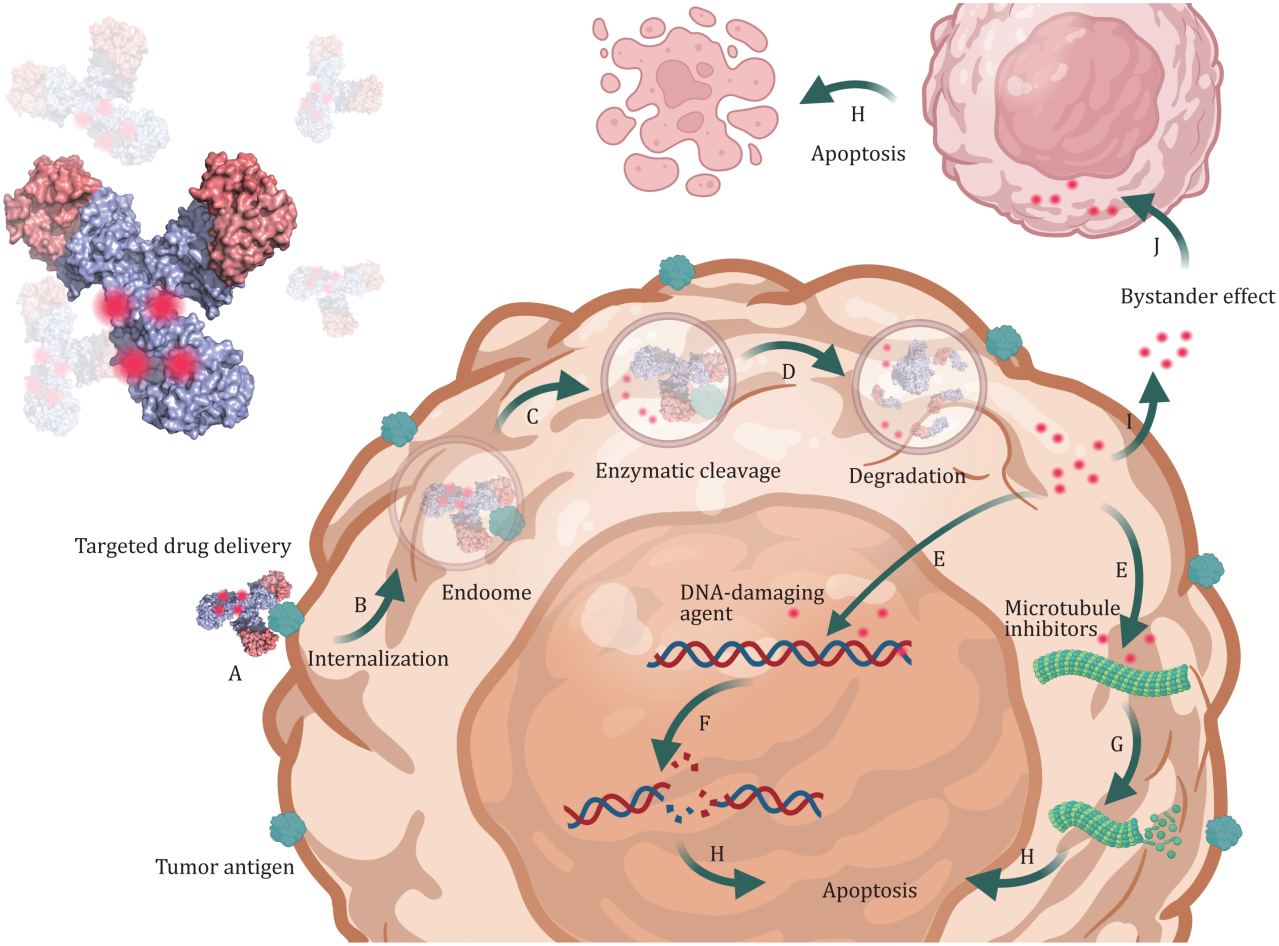
Mechanism of action of ADCs. (A) Antibody-antigen complexation occurs. (B) The complex undergoes endocytosis and lysosomal trafficking. (C) Lysosomal cathepsins cleave the ADC linker, liberating the cytotoxic payload.(D) Proteolytic degradation of the antibody ensues. (E) The payload targets DNA or microtubules. (F) DNA damage is induced. (G) Microtubule dynamics are disrupted. (H) Apoptosis of tumor cells. (I) The payload effluxes via membrane transporters or diffuses out if the membrane is permeable.(J) Membrane-permeable payloads induce a bystander effect on adjacent tumor cells.

ADCs are composed of three integral components: a targeting antibody, a biochemical linker, and a cytotoxic payload. The antibody facilitates the directed delivery of the ADCs due to its high affinity for specific antigens, while the linker modulates the binding affinity, stability, and release kinetics of the payload. The payload component is pivotal for the annihilation of tumor cells, and effective concentrations in the picomolar to nanomolar range are needed to exert these effects; payloads are primarily classified as either DNA-targeting or microtubule-targeting agents. Guided by antibodies, the delivery of the cytotoxic payload into tumor cells is achieved via antigen–antibody complex formation-mediated endocytosis (Fig. 1).

There have been three pivotal generational advances in the development of ADCs. Initially, ADCs were generated by coupling cytotoxic moieties to murine-derived antibodies, which were characterized by limited antigen specificity, via noncleavable linkers. These ADCs exhibited therapeutic potency that did not surpass that of unbound cytotoxic entities, compromised homogeneity, and significant potential immunogenicity (Ponziani et al., 2020). Subsequently, effective drugs were conjugated to humanized antibodies via acid-sensitive linkers, thereby enhancing the safety profile, and the first generation of ADCs, including gemtuzumab ozogamicin and inotuzumab ozogamicin, was granted market approval (Ricart, 2011). The limitations of first-generation ADCs, such as linker instability, propensity towards aggregation, and drug heterogeneity, were addressed with the advent of the second generation of ADCs, which was epitomized by brentuximab vedotin and ado-trastuzumab emtansine (Mei et al., 2014). This new generation used IgG1 subtype monoclonal antibodies with more toxic payloads, increasing both their solubility in water and conjugation efficiency. Furthermore, this new generation had markedly increased linker stability, resulting in a more homogeneous drug-to-antibody ratio (DAR) distribution (Beck et al., 2017). Despite the breakthroughs in therapeutic efficacy and safety achieved by the second-generation ADCs, challenges such as off-target toxicity, rapid clearance due to high DARs, and suboptimal therapeutic outcomes, remained. To address these challenges, third-generation ADCs, including polatuzumab vedotin, enfortumab vedotin, and fam-trastuzumab deruxtecan were developed with unified DAR values, significantly diminishing off-target toxicity and optimizing pharmacokinetic properties (Deeks, 2019; Narayan et al., 2021; Shitara et al., 2021). These advances were attributed to the use of fully humanized antibodies, which exhibit lower immunoreactivity, and the integration of novel linkers with improved hydrophilicity and stability, leading to reduced toxicity and increased anticancer efficacy, thus advancing ADC technology.

Although some ADCs have been approved by the FDA after exhibiting sufficient efficacy and safety profiles, the clinical administration of these therapeutics still results in a spectrum of substantial toxicities across different cancer patients. Indeed, adverse events are one of the principal reasons why many ADCs do not succeed in clinical trials (Endo et al., 2021; Nguyen et al., 2023; Zhu et al., 2023). The development of ADCs faces several challenges, including the complexity of their pharmacokinetics, insufficient tumor targeting and payload release, and toxicity issues (Nguyen et al., 2023). Among the 11 ADCs that have received global approval to date, 9 have been assigned a black box warning (Liu et al., 2022b; Tolcher et al., 2023). Although DS8201 (ENHERTU) was highly regarded for its efficacy across various cancers, it has been subject to safety concerns. For example, there have been reports of serious adverse effects, including interstitial lung disease (ILD) and pneumonia, and some of its adverse effects lead to fatal outcomes; embryonal-foetal toxicity has also been noted, indicating potential harm to the foetus if this drug is administered during pregnancy (Guo et al., 2022; Narayan et al., 2021).

## Marketed ADCs

As of November 2024, 11 ADCs have been successfully approved by the FDA, as detailed in Table 1. Among those, seven are indicated for the treatment of solid tumors, whereas four are for hematological malignancies. Although Cetuximab Sarotalocan (Akalux) and Disitamab Vedotin (RC48) have obtained approvals from the MHLW in Japan and the NMPA in China, respectively, they have not yet been approved by the FDA (Feng et al., 2024; Tanaka et al., 2023). In addition, belantamab mafodotin (Blenrep) was withdrawn from the market in 2022 due to failure to meet predefined efficacy endpoints in phase III clinical trials. However, new data from GSK’s phase III DREAMM-8 trial indicated that the Blenrep combination regimen significantly reduced the risk of disease progression or death by approximately 50% compared to standard therapy in patients with relapsed/refractory multiple myeloma, suggesting a potential market reentry (Dimopoulos et al., 2023; plc, 2024). Moxetumomab pasudotox (Lumoxiti), an antibody-conjugated immunotoxin, was voluntarily withdrawn from the market due to low sales. Gemtuzumab Ozogamicin (Mylotarg) was recalled in several key markets due to its association with severe veno-occlusive disease. Nonetheless, after dosage adjustment, Mylotarg was reapproved in 2017 for market entry (Norsworthy et al., 2018).

**Table 1.** FDA-approved ADCs.

No.	Product name	Drug name	Company	Target	Payload	Indications	Approval time	Black box warning
1	Mylotarg	Gemtuzumab Ozogamicin	Pfizer	CD33	Calicheamicin	AML	2017.9	Hepatotoxicity
2	Adcetris	Brentuximab Vedotin	Seagen/Takeda	CD30	MMAE	HL	2011.8	Progressive multifocal white brain disease
3	Kadcyla	Trastuzumab Emtansine	Roche/ImmunoGen	HER2	DM1	HER2+ BC	2013.2	Hepatotoxicity; cardiotoxicity; embryotoxicity
4	Besponsa	Inotuzumab ozogamicin	Pfizer	CD22	Calicheamicin	ALL	2017.8	Hepatotoxicity
5	Polivy	Polatuzumab Vedotin	Roche	CD79β	MMAE	DLBCL	2019.6	N/A
6	Padcev	Enfortumab Vedotin	Seagen/Astellas Pharma Inc.	Nectin-4	MMAE	la/mUC	2019.12	Severe skin toxicity
7	Enhertu	Trastuzumab deruxtecan	Daiichi Sankyo Company Limited/Astrazeneca	HER2	Dxd	HER2+ BC HER2+ GC	2019.12	Interstitial lung disease; embryotoxicity
8	Trodelvy	Sacituzumab govitecan	Immunomedics	Trop2	SN38	TNBC	2020.4	Neutropenia; diarrhoea
9	Zynlonta	Loncastuximab tesirine	ADC Therapeutics	CD19	PBD	DLBCL	2021.4	N/A
10	Tivdak	Tisotumab Vedotin	Seagen	TF	MMAE	CC	2021.9	Ocular toxicity
11	Elahere	Mirvetuximab soravtansinegynx	Zhongmeihuadong Pharmaceutical Co., Ltd./ImmunoGen	FRα	DM4	PROC	2022.11	Ocular toxicity

**Abbreviations:** AML, acute myeloid leukaemia; HL, Hodgkin lymphoma; HER2+, human epidermal growth factor receptor 2 positive; BC, breast cancer; ALL, acute lymphoblastic leukaemia; HCL, hairy cell leukaemia; DLBCL, diffuse large B-cell lymphoma; la/mUC, locally advanced or metastatic urothelial carcinoma; GC, gastric cancer; TNBC, triple-negative breast cancer; MM, multiple myeloma; HNC, head and neck cancer; CC, cervical cancer; PROC, platinum-resistant ovarian cancer; BLC, bladder cancer.

Notably, following Pfizer’s $43 billion acquisition of Seagen and AbbVie’s $10.1 billion purchase of ImmunoGen in 2023, the ADC sector has maintained strong momentum into 2024, highlighted by significant transactions such as Johnson & Johnson’s $2 billion acquisition of Ambrx Biopharma and Genmab’s $1.8 billion takeover of ProfoundBio. These strategic investments are motivated by the clinical and commercial success of ADC therapeutics, as exemplified by AstraZeneca and Daiichi Sankyo’s Enhertu, which generated over $2.5 billion in revenue in 2023. Moreover, Chinese companies, like MediLink Therapeutics have emerged as key players in the global ADC landscape, advancing the sector through technological innovation and international collaboration (Kirkpatrick, 2024).

## ADCs that have failed in phase I, II, or III clinical trials

**Figure 2.**
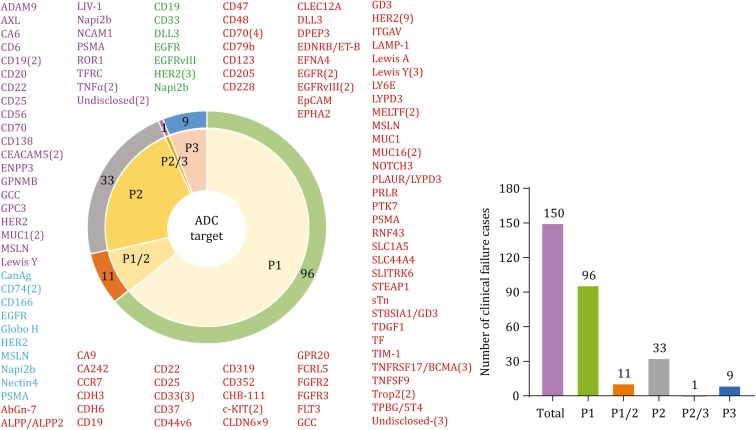
Antigen targets of clinically tested ADCs. The following shows the number of ADCs that failed in phases I-III clinical trials and their corresponding stage distribution.

Based on comprehensive database analyses and recent systematic reviews, a total of 267 ADCs are currently under clinical evaluation worldwide (Dumontet et al., 2023; Flynn et al., 2024). The therapeutic application of more than 80% (approximately 225 ADCs) of these drugs in the treatment of solid tumors is being studied, highlighting the pivotal role of ADC drugs in the treatment of solid tumors. The targets of ADCs that have failed at different stages of clinical trials are shown in Fig. 2. Additionally, a list of the majority of ADCs that have failed in phase I/II/III clinical trials for cancer treatment to date, is presented in Table 2.

**Table 2.** Sources and indications of ADCs that failed clinical trials.

No.	Target	Drug name	The first R&D enterprise	Indications	The world’s highest research and development stage
1	AbGn-7	AbGn-107	AbGenomics B.V	STAD; COADREAD; PAAD; BTC	Terminated phase 1
2	ADAM9	IMGC-936	ImmunoGen, Inc.	SC; NSCLC; m/COADREAD; TNBC; EC; STAD; mSTAD; PAAD; mNSCLC; MEC; mPC	Terminated phase 2
3	ALPP; ALPPL2	SGN-ALPV	Seagen Inc.	SC	Terminated phase 1
4	AXL	AXL-107-MMAE	Genmab	UCEC; MEL; NSCLC; OV; Sarcoma; TC; CC	Terminated phase 2
5	CA6	SAR-566658	ImmunoGen, Inc.	SC; mBC	Terminated phase 2
6	CA9	BAY79-4620	Bayer Pharma AG	SC	Terminated phase 1
7	CA242	PNU 214565	Pharmacia & Upjohn	COADREAD; PAAD	Terminated phase 1
8	CanAg	C-242-DM1	ImmunoGen, Inc.	COAD; mCOAD; NSCLC; PAAD; STAD; RPMM; MOC	Terminated phase 1/2
9	CCR7	JBH-492	Novartis Pharmaceuticals Corp	NHL; Recurrent CLL	Terminated phase 1
10	CDH3	PCA062	ImmunoGen, Inc.; Novartis Pharmaceuticals	TNBC; HNC; UCEC	Terminated phase 1
11	CDH6	HKT-288	Novartis Pharmaceuticals	EOC; RCC	Terminated phase 1
12	CD6	Oncolysin CD6	ImmunoGen, Inc.	BC; Leukaemias; Lymphoma; TR; AD; AID	Terminated phase 2
13	CD19	Denintuzumab mafodotin; SGN-CD19A	Seagen Inc.	BCL; DLBCL; FL, Grade 3b	Terminated phase 2
14		SGN CD19B	Seagen Inc.	DLBCL; FL; Refractory Aggressive NHL	Terminated phase 1
15		Coltuximab ravtansine; SAR-3419	Sanofi	ALL	Terminated phase 2
16		ANTI-B4-BR; Oncolysin B	Dana Farber Cancer Institute Inc.	MN; BMT; Lymphoma; SCLC	Terminated phase 3
17	CD20	MT-3724	Molecular Templates Inc.	CLL; DLBCL; FL; HNL MCL; NHL	Terminated phase 2
18	CD22	BAY-1862864; CD22-TTC	Bayer Pharma AG	HNL; NHL	Terminated phase 1
19		RG-7593	Genentech, Inc.	DLBCL; FL; HNL	Terminated phase 2
20	CD25	ADCT-301 (camidanlumab tesirine)	ADCT-301	AML; MDS; MPN	Terminated phase 2
21		RM-1995	Rakuten Medical, Inc.	CSCC; HNSCC	Terminated phase 1
22	CD33	IMGN-779	ImmunoGen, Inc.	APML	Terminated phase 1
23		AVE 9633	ImmunoGen, Inc.	AML	Terminated phase 1
24		AVE-9633	ImmunoGen, Inc.	Relapsed/Refractory CD33 + AML	Terminated phase 1
25		SGD-1882; SGN-CD33A	Seagen Inc.	APML; AML	Terminated phase 3
26	CD37	AGS67E	Astellas Pharma Inc.; Seagen Inc.	LM; AML	Terminated phase 1
27	CD44v6	B1W1-1	Boehringer Ingelheim; ImmunoGen, Inc.	HNSCC	Terminated phase 1
28	CD47	SGN-CD47M	Seagen Inc.	STS; COADREAD; HNSCC; NSCLC; BC; OV; Exocrine PAAD	Terminated phase 1
29	CD48	SGN-CD48A	Seagen Inc.	MM	Terminated phase 1
30	CD56	N901-bR	ImmunoGen, Inc.	SCLC	Terminated phase 2
31	CD70	SGN-75	Seagen Inc.	RCC; NHL	Terminated phase 1
32		SGN-CD70A	Seagen Inc.	READ; MCL; DLBCL; FL	Terminated phase 2
33		MDX-1203	Bristol-MyersSquibb; Medarex	RCC; NHL	Terminated phase 1
34		MDX-1411	Medarex Inc.	MN	Terminated phase 1
35		AMG 172	Amgen; ImmunoGen, Inc.	RCC	Terminated phase 1
36	CD74	IMMU-110	Immunomedics, Inc.	NHL; CLL	Terminated phase 1/2
37		BN-301	Sutro Biopharma Inc.	Advanced BCM	Terminated phase 1/2
38	CD79b	RG-7986	Genentech, Inc.; Seagen Inc.	NHL	Terminated phase 1
39	CD123	SGN-CD123A	Seagen Inc.	AML	Terminated phase 1
40	CD138	Indatuximab ravtansine	Biotest Pharma GmbH	Relapsed/Refractory MM	Terminated phase 2
41	CD166	CX-2009	ImmunoGen, Inc.	BC; NSCLC; HNC; OV	Terminated phase 1/2
42	CD205	MEN-1309	Menarini Group	Metastatic SC; R/R NHL	Terminated phase 1
43	CD228	SGN-CD228A	Seagen Inc.	MCM; MPM; HER2– BC; NSCLC; PDAC; COADREAD	Terminated phase 1
44	CD319	ABBV-838	AbbVie, Inc.	MDS	Terminated phase 1
45	CD352	SGN-CD352A	Ligand Pharmaceuticals, Inc.	MM	Terminated phase 1
46	CEACAM5	IMMU-130	Immunomedics, Inc.	COADREAD	Terminated phase 2
47		SAR408701	ImmunoGen, Inc.	Advanced SC	Terminated phase 2
48	CHB-111	CHB 111	ViRexx Medical Corp.	Hepatitis B	Terminated phase 1
49	c-KIT	LOP-628	Novartis Pharmaceuticals	cKIT+ SC; AML	Terminated phase 1
50		CD117-ADC	Magenta Therapeutics, Inc.	AML; MDS	Terminated phase 1
51	CLDN6 × 9	SC-004	AbbVie; Stemcentrx	EOC; UCEC	Terminated phase 1
52	CLEC12A	RG-6109	Genentech, Inc.	AML	Terminated phase 1
53	DLL3	rovalpituzumab tesirine	Stemcentrx Inc.	SCLC	Terminated phase 3
54		SC-002	AbbVie; Stemcentrx	SCLC	Terminated phase 1
55	DPEP3	SC-003	AbbVie, Inc.	NSCLC	Terminated phase 1
56	EDNRB; ET-B	RG7636	Genentech, Inc.	MEL;	Terminated phase 1
57	EFNA4	PF-0667263	Pfizer	TNBC	Terminated phase 1
58	EGFR	ABBV-221	AbbVie	HNSCC; NSCLC; TNBC; COADREAD; GBM	Terminated phase 1
59		AVID100	Formation Biologics	SC; TNBC; HNSCC; NSCLC	Terminated phase 1/2
60		Depatuxizumab mafodotin; ABT-414	AbbVie	GBM	Terminated phase 3
61		IMGN289	ImmunoGen, Inc.	EGFR+ SC	Terminated phase 1
62	EGFRvIII	ABT-414	Abbott Laboratories	GBM; AA	Terminated phase 3
63		AMG-595	Amgen Inc.	GBM	Terminated phase 1
64		ABBV-321	AbbVie Inc.	Advanced SC	Terminated phase 1
65	ENPP3	AGS-16M8F	Agensys Inc.	mRCC	Terminated phase 2
66	EpCAM	LY-256787	Eli Lilly & Co.	AAC	Terminated phase 1
67	EPHA2	MEDI 547;MI-CP177	AstraZeneca; Seagen Inc.	SC	Terminated phase 1
68	FCRL5	RG-7598	Genentech, Inc.; Seagen Inc.	MM	Terminated phase 1
69	FGFR2	BAY1187982	Bayer	FGFR2+ SC	Terminated phase 1
70	FGFR3	LY-3076226	Eli Lilly and Company; ImmunoGen, Inc.	SC	Terminated phase 1
71	FLT3	AGS-62P1;ASP-1235	Ambrx, Inc.	APML	Terminated phase 1
72	GCC	indusatumab vedotin;MLN-0264	Millennium Pharmaceuticals Ltd; Seagen Inc.	SC; GIC; PAAD; GEJC	Terminated phase 2
73		TAK-164	ImmunoGen, Inc.	COADREAD; GIC; mCOADREAD; PAAD; STAD	Terminated phase 1
74	GD3	PF-06688992	Pfizer	Stage IV MEL	Terminated phase 1
75	Globo H	OBI-999	OBI Pharma Inc.	Advanced SC	Terminated phase 1/2
76	GPC3	BMS-986183	Bristol Myers Squibb Co.	LC	Terminated phase 2
77	GPNMB	CDX-011	Celldex Therapeutic; Seagen Inc.	TNBC; SCLC; ROS; RUM; Stage IV UVM	Terminated phase 2
78	GPR20	DS-6157a	Daiichi Sankyo, Inc.; Sarah Cannon Research Institute	GIST	Terminated phase 1
79	HER2	PF-06804103	Pfizer	SC; BBC; NSCLC; STAD	Terminated phase 1
80		AZ-13599185;MEDI-4276	AstraZeneca	SC; BBC; STAD	Terminated phase 1
81		BAY-2701439	Bayer Pharma AG	SC; MBC; MEM; mSTAD	Terminated phase 1
82		XMT-1522;TAK-522	Mersana Therapeutics Inc.	BBC; NSCLC; STAD	Terminated phase 1
83		BAT-8001	Bio-Thera Solutions	HER2+ ABC; HER2+ GG	Terminated phase 3
84		TAA-013;TAT-013	Tot Biopharm	SC; MBC	Terminated phase 3
85		YO28405	Hoffmann-La Roche	MBC	Terminated phase 3
86		ADCT-502	ADC Therapeutics S.A.	HER2+ BC; NSCLC; GEC; BLC	Terminated phase 1
87		RG6148	Genentech, Inc.	BC	Terminated phase 1
88		NJH395	Novartis	Nonbreast HER2+ malignancies	Terminated phase 1
89		SBT6065	Silverback Therapeutics	HER2+ SC	Terminated phase 1/2
90		BI-CON-02	BioIntegrator LLC	HER2+ MBC	Terminated phase 1
91		MM-302	Merrimack Pharmaceuticals, Inc.	ABC; HER2+ ABC; HER2+ BC; BC	Terminated phase 2/3
92		BDC-1001	Bolt Biotherapeutics Inc.	COADREAD; MBC; mSTAD; UCEC	Terminated phase 2
93		ZW49	Zymeworks Inc.	HER2+ cancers	Terminated phase 1
94	ITGAV	IMGN388	ImmunoGen, Inc.	SC	Terminated phase 1
95	LAMP-1	SAR428926	ImmunoGen, Inc.; Sanofi	SC	Terminated phase 1
96	Lewis-A	AbGn-7	AbGenomics International, Inc.	AMSN; GEN; RmSTAD; STAD	Terminated phase 1
97	Lewis Y	CMD-193	Wyeth; Pfizer	SC	Terminated phase 1
98		SGN-15	Bristol-Myers Squibb Co.	RBC; CRPC; COADREAD; NSCLC; OV; PAAD RNSCLC	Terminated phase 2
99		GNX-102	GlycoNex, Inc.	AMSN	Terminated phase 1
100	LIV-1	MK-6440 (Ladiratuzumab vedotin)	Seagen Inc.	Advanced SC	Terminated phase 2
101	LY6E	RG7841	Genentech, Inc.; Seagen Inc.	SC; BC; NSCLC	Terminated phase 1
102	LYPD3	Lupartumab amadotin	Bayer AG	AMSN	Terminated phase 1
103	MELTF	SGN-CD228A	Seagen Inc.	CM; PM; HER2–BC; NSCLC; COADREAD; PDAC	Terminated phase 1
104		SC-005	Abbvie; Stencentrx	TNBC	Terminated phase 1
105	MSLN	BAY-94-9343	Bayer Pharma AG	SC; MESO; PMM; OV	Terminated phase 2
106		MDX-1204;BMS-986148	Bristol-MyersSquibb; Medarex	MESO; NSCLC; OV; PAAD; STAD	Terminated phase 1/2
107		RG7600	Genentech, Inc.; Seagen Inc.	OV; PAAD	Terminated phase 1
108	MUC1	CMB-401	Pfizer; Celltech	OV; PAAD	Terminated phase 2
109		SB-408075	GlaxoSmithKline; ImmunoGen, Inc.	NSCLC; COADREAD; PAAD	Terminated phase 1
110		IMGN242	ImmunoGen, Inc.	SN; STAD; GEJC	Terminated phase 2
111	MUC16	RG-7882	Genentech, Inc.	OV; PAAD	Terminated phase 1
112		RG-7458	Genentech, Inc.	OV	Terminated phase 1
113	Napi2b	RG-7599	Genentech, Inc.	NSCLC; OV	Terminated phase 2
114		XMT-1592	Mersana Therapeutics; Synaffix	OV; NSCLC	Terminated phase 1/2
115		XMT-1536	Mersana Therapeutics Inc.	PN; FTC; MOC; TC; RCC; SGC; NSCLC; MN	Terminated phase 3
116	NCAM1	IMGN-901	ImmunoGen, Inc.	SC; SCLC; MM; MCC	Terminated phase 2
117	Nectin4	SBT6290-101	ARS Pharmaceuticals; Silverback Therapeutics	BLCA; TNBC; NSCLC; HNSCC; HR+/HER2− BC	Terminated phase 1/2
118	NOTCH3	PF-06650808	Pfizer	SC; BC	Terminated phase 1
119	PLAUR; LYPD3	BAY1129980	Bayer; Seagen Inc.	NEO	Terminated phase 1
120	PRLR	ABBV-176	AbbVie	Advanced solid tumors likely to express PRLR	Terminated phase 1
121	PTK7	PF-7020	AbbVie	SC; NSCLC	Terminated phase 1
122	PSMA	MLN2704	Millennium Pharmaceuticals, Inc.	PCa	Terminated phase 1/2
123		PSMA ADC (Progenics Pharmaceuticals)	Progenics Pharmaceuticals, Inc.	mCRPC	Terminated phase 2
124		MEDI-3726	ADC Therapeutics S.A.; AstraZeneca	mCRPC	Terminated phase 1
125	RNF43	SC-006	AbbVie, Inc.	Advanced SC	Terminated phase 1
126	ROR1	NBE-002	NBE-Therapeutics AG	Advanced SC	Terminated phase 2
127	SLC1A5	MEDI-7247	AstraZeneca	mCOAD; HLT; HRPC; PAAD; NSCLC; SCLC	Terminated phase 1
128	SLC44A4	ASG-5ME	Agensys Inc.	PAAD; STAD	Terminated phase 1
129	SLITRK6	ASG-15ME	Agensys Inc.	BLC; TCP	Terminated phase 1
130	STEAP1	RG-7450	Genentech, Inc.; Seagen Inc.	PCa	Terminated phase 1
131	sTn	SGN-STNV	Seagen Inc.	AMSN	Terminated phase 1
132	ST8SIA1; GD3	PF-06688992	Pfizer	MEL	Terminated phase 1
133	TDGF1	BIIB015	Biogen Idec; ImmunoGen, Inc.	SC	Discontinued phase 1
134	TF	ICON-2;XB 002	Iconic Therapeutics, Inc.	AFP + SC; UCEC; ESCC; HR+ BC; mCRPC; NSCLC; EOC; PAAD; HNSCC; TNBC; CC	Terminated phase 1
135	TFRC	CX-2029	CytomX Therapeutics Inc.	SCC; DLBCL; ASC; mNSCLC; MEC; mHNSCC; MGM; UCEC	Terminated phase 2
136	TIM-1	CDX-014; CR-012; CR-014	CuraGen Corp	RPMM; MOC	Terminated phase 1
137	TNFRSF17 BCMA	AMG224	Amgen	MM	Terminated phase 1
138		MEDI-2228	AstraZeneca	MM	Terminated phase 1
139		CC-9971	Sutro Biopharma Inc.	Relapsed/Refractory MM	Terminated phase 1
140	TNFSF9	SC-007	Abbvie; Stemcentrx	COADREAD; STAD	Terminated phase 1
141	TNFα	ABBV-154	AbbVie Inc.	CD; RA; PMR	Terminated phase 2
142		ABBV-3373	AbbVie Inc.	RA; IBD	Terminated phase 2
143	Trop2	BAT-8003	Bio-Thera Solutions	BLC; MBC; mNSCLC	Terminated phase 1
144		PF-06664178	Pfizer	SC; BLM	Terminated phase 1
145	TPBG/5T4	PF-06263507	Pfizer	NEO; NSCLC; BC; OV	Terminated phase 1
146	-	Immunoconjugate (Bayer AG)	Bayer AG	NEO	Terminated phase 1
147	-	BIO111	SWITCH ELECTRIC LTD	BLC	Terminated phase 1
148	-	B cell High Density Microparticles	-	Lymphoma	Terminated phase 2
149	-	ZD-2767	AstraZeneca plc	COADREAD; SC	Terminated phase 2
150	-	RG-7861	Symphogen A/S	S. aureus; ID	Terminated phase 1

**Abbreviations:** SC, solid carcinoma; AFP + SC, AFP expression solid carcinoma; AAC, advanced adenocarcinoma; BBT, benign breast carcinoma; NSCLC, non-small cell lung cancer; STAD, stomach adenocarcinoma; MBC, metastatic breast carcinoma; MEM, metastatic oesophageal malignancy; mSTAD, metastatic gastric cancer; RmSTAD, recurrent metastatic gastric carcinoma; BC, breast carcinoma; HER2+, human epidermal growth factor receptor 2 positive; ABC, advanced breast carcinoma; RBC, recurrent breast carcinoma; GEC, gastroesophageal cancer; HNSCC, head and neck squamous cell carcinoma; SCC, squamous cell carcinoma; CSCC, cutaneous squamous cell carcinoma; TNBC, triple-negative breast carcinoma; COADREAD, colon adenocarcinoma/rectum adenocarcinoma esophageal carcinoma; GBM, glioblastoma multiforme; EGFR+, epidermal growth factor receptor positive; AA, anaplastic astrocytoma; mNSCLC, metastatic non-small cell lung cancer; RNSCLC: recurrent NSCLC; BLM, bronchial or lung malignancy; LC, liver cancer; OV, ovarian carcinoma; PAAD, pancreatic adenocarcinoma; CA242+, CA242 positive; SN, stomach neoplasms; GEJC, gastroesophageal junction cancer; BLCA, bladder urothelial carcinoma; HR+, hormone receptor positive; MESO, mesothelioma; NEO, neoplasms; CC, cervical cancer; UCEC, uterine corpus endometrial carcinoma; EC: embryonal carcinoma; ESCC, esophageal squamous cell carcinoma; MEL, melanoma; MM, multiple myeloma; RMM, relapse multiple myeloma; MEC, metastatic esophageal cancer; AMSN, advanced malignant solid neoplasm; GEN: glandular and epithelial neoplasms; GIC, gastrointestinal carcinoma; TC, thyroid carcinoma; EOC, epirhelial; CM, cutaneous melanoma; PM, pleural mesothelioma; PDAC, pancreatic ductal adenocarcinoma; PMM, pancreatic metastatic malignancy; MOC, malignant ovarian carcinoma; mCOAD, malignant colon adenocarcinoma; MCC, merkel cell carcinoma; MN, malignant neoplasm; PN, peritoneal neoplasm; FTC, fallopian tube carcinoma; SGC, salivary gland carcinoma; UVM, uveal melanoma; PRL-R, prolactin receptor; RPMM, renal or renal pelvis metastatic malignancy; HLT, hematopoietic or lymphoid tissue; HRPC, hormone-resistant prostate carcinoma; SCLC, small cell lung cancer; PCa, prostate carcinoma; mCRPC, metastatic castration-resistant prostate cancer; BTC, biliary tract cancer; HNC, head and neck cancer; EOC, epithelial ovarian carcinoma; RCC, renal cell carcinoma; ROC, recurrent osteosarcoma; RUM, recurrent uveal melanoma; GIC, gastrointestinal cancer; mRCC, metastatic renal cell carcinoma; BLC, bladder cancer; TCP, transitional cell papilloma; FGFR2+, fibroblast growth factor receptor 2 positive; GIST, gastrointestinal stromal tumor; AML, acute myeloid leukaemia; cKIT+, vKIT positive; MDS, myelodysplastic syndromes; BCL, B-cell lymphoma; DLBCL, diffuse large B-cell lymphoma; FL, follicular lymphoma; ALL, acute lymphocytic leukaemia; CLL, chronic lymphocytic leukaemia; HNL, hematopoietic and lymphoid neoplasms; MCL, mantle cell lymphoma; NHL, non-hodgkin lymphoma; MPN, myeloproliferative neoplasm; GCC, germ cell carcinoma; TC, testicular carcinoma; NGCC, nonseminomatous germ cell carcinoma; HL, Hodgkin lymphoma; DLBCL-R, diffuse large B-cell lymphoma refractory; APML, acute myeloid leukaemia and related precursor neoplasms; LM, lymphoid malignancy; STS, soft tissue sarcoma; READ, rectum adenocarcinoma; R/RR NHL, relapsed/refractory non-Hodgkin lymphoma; BCM, B-cell malignancies; CD, Crohn’s disease; RA, rheumatoid arthritis; PMR, polymyalgia rheumatical; IBD, inflammatory bowel disease; TR, transplant rejection; AD, autoimmune disease; AID, autoinflammatory disorders; MCM, metastatic cutaneous melanoma; MPM, malignant pleural mesothelioma; *S. aureus*, *Staphylococcus aureus* infection; ID, infectious disease(s); BMT, bone marrow transplantation.

The table excludes information trials of ADCs combined with other drugs. Based on updated and revised data as of November 2024, there were 150 instances where monotherapy with ADCs failed during phase I to III clinical trials; 37 were evaluated in trials on haematological malignancies (37/150, 24.7%), and 114 were evaluated in trials on solid tumors (114/150, 76%) (Colombo et al., 2024). Specifically, 96 ADC treatments failed in phase I clinical trials (96/150, 64%), including one case that was discontinued during the phase I study, 11 failed in phase I/II clinical trials (11/150, 7.3%), 33 that failed in phase II clinical trials (33/150, 22%), 1 that failed in phase II/III clinical trials (1/150, 0.67/%), and 9 that failed in phase III clinical trials (9/150, 6%). Among the solid tumor receptors that are targeted by these ADCs, HER2 is predominant, followed by EGFR, Trop2, MUC-1, Nectin4, CEACAM5, MSLN, 5T4, AXL, etc. For haematological malignancies, the primary targets are CD19, CD22, CD30, CD33, CD70 and KIT.

The divergent outcomes observed in ADCs targeting identical antigens stem from a complex interplay of factors, underscoring the multifaceted nature of ADC development. At the molecular level, successful ADCs typically exhibit superior antibody characteristics, including optimal affinity, specificity, and internalization kinetics, which synergize with meticulously tailored linker technology whose stability and cleavage properties are crucial for intracellular efficacy. The pharmacological profile is further shaped by the payload’s potency, membrane permeability, and solubility, significantly influencing both efficacy and pharmacokinetics, whereas the drug-antibody ratio (DAR) necessitates a delicate equilibrium between potency and stability. From a developmental perspective, manufacturing consistency and scalability are paramount for regulatory approval and successful commercialization, complemented by strategic elements in clinical trial design, particularly patient stratification based on robust biomarker strategies and optimized dosing regimens. Concurrently, resistance mechanisms, such as drug efflux and epigenetic alterations, pose persistent challenges, and the safety profile, encompassing the therapeutic window and off-target effects, often distinguishes successful candidates. Beyond these scientific considerations, corporate resources and expertise in ADC development can be decisive factors in navigating the complex landscape of drug development. Perhaps most fundamentally, the biological characteristics of the target antigen—including expression levels, internalization rates, and potential for antigen drift—profoundly influence ADC performance and serve as the cornerstone for successful development.

Among these factors, target specificity is paramount, as it directly influences both the efficacy and safety of ADCs. This specificity hinges primarily on the antigen recognized by the antibody, rendering the selection of appropriate antigens a critical challenge in ADC development. Ideal ADC targets should exhibit high expression on tumor cells while maintaining minimal or no expression in normal tissues. However, identifying such “perfect” antigens has proven to be exceptionally challenging. Numerous antigens initially presumed to be tumor-specific have subsequently been detected in certain normal tissues, often leading to off-target toxicity. Moreover, intratumoral heterogeneity and temporal fluctuations in antigen expression further complicate target selection. Consequently, advancing our understanding of tumor biology and antigen expression dynamics, coupled with the development of more sophisticated targeting strategies, is crucial for enhancing the therapeutic index of ADCs. This multifaceted approach may pave the way for the next generation of ADCs with improved efficacy and safety profiles.

## Target selection

**Figure 3.**
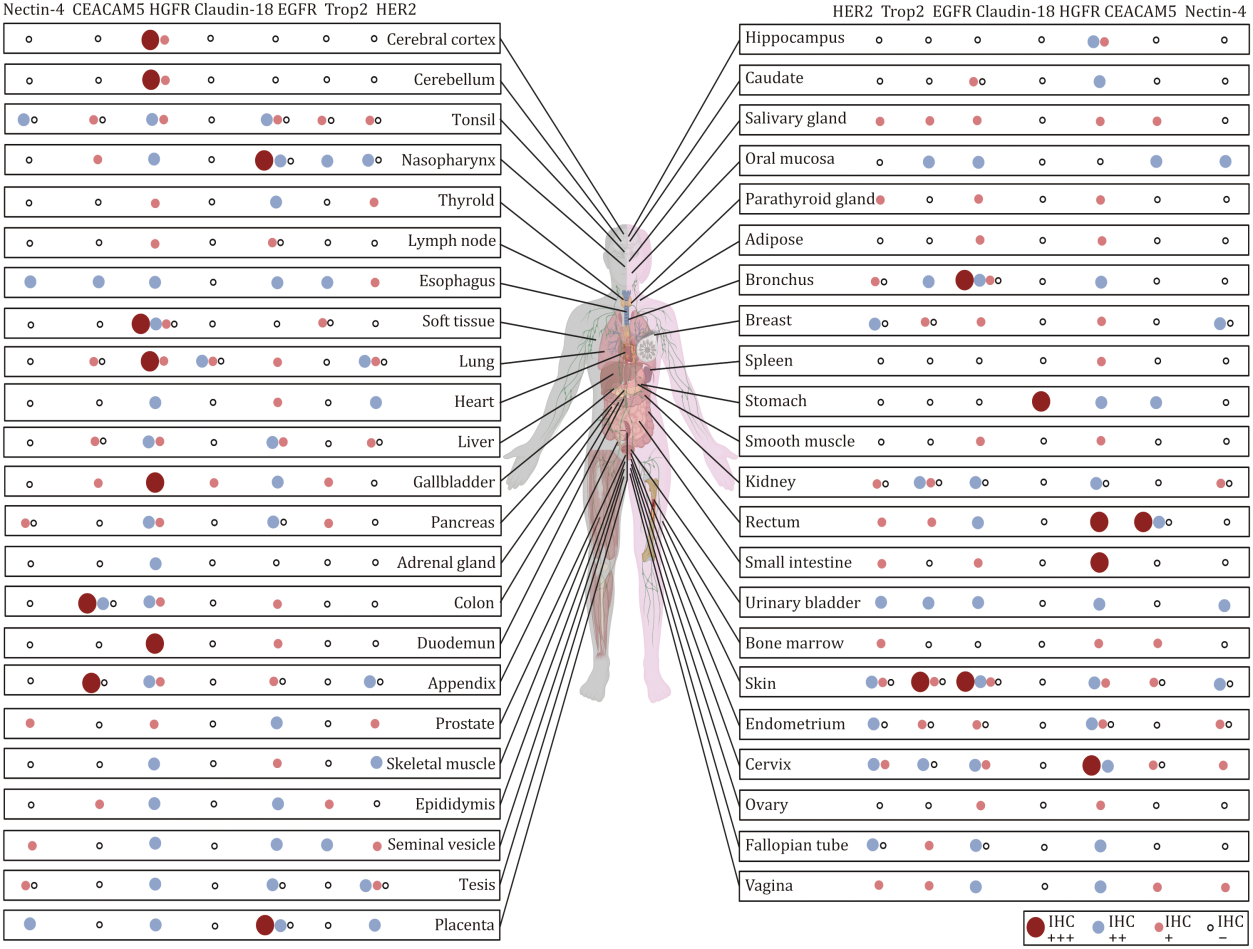
The tissues analysed in this study include only those studied by antibody-based profiling. In the immunohistochemistry results, protein expression levels are represented as follows: (**+++**) indicate high expression; (**++**) denote moderate expression; (**+**) represent low expression; and (–) signify undetectable levels. The size of the circles correlates with the relative expression level of the corresponding protein, with larger circles indicating higher expression. The figure above involved 144 individuals from whom 44 distinct types of normal tissues were collected. To ensure data representativeness, tissue samples for immunohistochemical analysis were sourced from multiple individuals. The majority of normal tissue types were represented by three samples, each derived from a tissue core of a distinct individual. However, to account for potential variability, endometrial, skin, soft tissue, and gastric samples were each collected from six individuals. Due to limitations in tissue availability, the parathyroid was represented by a single sample. This sampling strategy aimed to balance comprehensive coverage with the practical constraints of human tissue acquisition. Note: Protein expression levels across different targets should not be directly compared. For instance, immunohistochemistry (IHC) showing moderate Trop2 expression and high Met expression does not imply that Met is expressed at higher levels than Trop2. Such comparisons can be misleading due to variations in protein characteristics, antibody affinities, and detection methodologies.

Understanding the distribution and expression of target proteins in normal human tissues is key to predicting the adverse effects of ADCs. This knowledge forms a foundation for evaluating drug targets and foreseeing potential side effects. By mapping protein expression patterns, researchers can anticipate safety issues and develop mitigation strategies, thus improving the safety and effectiveness of targeted therapies. This summary highlights major target distributions across human tissues and organs. Notably, FRα and TF data are absent from The Human Protein Atlas whereas the distribution of other described targets is shown in Fig. 3. Enhancing protein atlases is crucial for advancing drug design and developing safer therapies.

### HER2

Human epidermal growth factor receptor 2 (HER2, also known as ERBB2) belongs to the epidermal growth factor receptor family and plays critical roles in several cellular processes, including cell proliferation (Amisha et al., 2023). The HER2 protein is ubiquitously expressed across a wide range of normal tissues, including the skin, oral mucosa, breasts, ovaries, uterine lining, lungs, pancreas, small and large intestines, kidneys, bladder, heart, central nervous system, and epithelial cell membranes of the placenta. Notably, HER2 protein levels are significantly elevated in foetal tissues compared with the corresponding tissues in normal adults (Alrhmoun and Sennikov, 2022). In the context of oncology, notable HER2 overexpression has been documented in cases of breast, gastric and ovarian cancers, indicating its relevance in the pathophysiology and its potential value as a therapeutic target of these cancers (Hofheinz et al., 2023; Kim et al., 2024). As a diagnostic marker for lung cancer, HER2 overexpression is predominantly observed in adenocarcinomas and large cell carcinomas (LCCs), with a reduced frequency in non-small cell lung cancer (NSCLC) tumors (Abu Al Karsaneh et al., 2023). A significant proportion of colorectal cancers also exhibit positive HER2 expression. Conversely, the prevalence of HER2 overexpression is decreased in cases of uterine, pancreatic, and head and neck cancers, and HER2 is generally not expressed in kidney or liver cancers, illustrating a complex pattern of expression that affects therapeutic decisions and outcomes across various cancer types (Hsu et al., 2002; Randall et al., 2024; Wang et al., 2012, 2020a; Warren et al., 2021).

Among ADCs that have reached commercialization and are currently in the research phase, most ADCs target HER2. Three such ADCs have been approved for the market: trastuzumab emtansine, trastuzumab deruxtecan, and disitamab vedotin. As shown in Table 1, among these three HER2-targeting ADCs that are available on the market, trastuzumab emtansine and trastuzumab deruxtecan have black box warnings due to their serious adverse effects. Trastuzumab emtansine is indicated for patients with residual HER2-positive tumor cells after neoadjuvant treatment, and its warning is related to hepatoxicity, cardiotoxicity, and embryotoxicity (Poon et al., 2013; von Minckwitz et al., 2019). Trastuzumab deruxtecan is indicated for patients with inoperable or metastatic HER2-positive breast cancer who have previously undergone at least two anti-HER2 therapy regimens, and its black box warnings are related to interstitial lung disease and embryotoxicity (Abuhelwa et al., 2022; Hacker, 2020; Keam, 2020). Disitamab vedotin is indicated for patients with HER2-overexpressing locally advanced or metastatic gastric cancer (including gastroesophageal junction adenocarcinoma) who have received at least two prior systemic therapies (Deeks, 2021). According to a meta-analysis that included data from 15 studies and 4,246 patients, the estimated incidence of fatal adverse events among patients treated with HER2-targeting ADCs is 0.78%. According to the available data, the most common fatal adverse event associated with HER2-targeting ADCs is respiratory toxicity, which accounted for 16 deaths, representing 37.0% of all studied fatalities. These cases included six cases of pneumonia and three cases of pneumonitis, along with one case each of interstitial lung disease, pulmonary embolism, aspiration pneumonia, lung infection, bronchopneumonia, dyspnoea, and atypical pneumonia. Haematotoxicity is the second most common adverse event associated with HER2-targeting ADCs, and this haematotoxicity caused nine deaths, representing 20.9% of the total studied fatalities. Other less common fatal adverse events include infections and hepatotoxicity (Fu et al., 2023). The data presented above indicate that although the use of HER2 as a therapeutic target has shown success in clinical trials and received market approval, its widespread expression in normal tissues and critical biological functions may result in severe adverse effects. This highlights the necessity of further in-depth research on HER2-targeted therapies, providing additional focal points for future scientific investigations.

It has long been known that HER2 is expressed in the heart, albeit at limited levels. The discovery of cardiac toxicity associated with HER2-targeting ADC therapies has spurred new research, which has revealed that the HER2 receptors that are expressed on adult cardiac muscle cell membranes play crucial roles in transmitting growth and survival signals (Slavcheva and Angelov, 2023). Upon binding by its ligand, neuregulin-1, HER2 forms heterodimers, initiating pathways that promote cell growth and survival. These activated pathways include the phosphoinositide 3-kinase, protein kinase A, and mitogen-activated protein kinase pathways (Wu et al., 2022a). Given the relatively low level of HER2 protein expression in the heart, it appears that there is no direct link to cardiac toxicity. However, the expression of HER2, coupled with its vital function in normal tissue and organ systems, may contribute to the severe adverse reactions observed after treatment with HER2-targeting ADC drugs. These reactions can include cardiac toxicity, embryotoxicity, and interstitial lung disease, indicating the nuanced risks associated with targeting HER2 in cancer treatment. In conclusion, the low expression of HER2 in cardiac tissues may lead to cardiotoxicity after the administration targeted therapies. This potential risk should be carefully considered in the design and development of future ADCs.

### Trop2

Trophoblast cell surface antigen 2 (Trop2) is a cell surface glycoprotein that plays an essential role in human embryonic development. Trop2 is ubiquitously expressed across a wide range of normal tissues, including the stratified squamous epithelium of the cervix, skin, oesophagus, and crypts of the tonsils. Trop2 is also a marker of cuboidal or columnar epithelia found in the breasts and prostate, uterine glands and endometrium, bile ducts, distal tubules, renal collecting ducts, and glandular and tubular epithelia of the salivary glands and pancreas (Qiu et al., 2023). Furthermore, Trop2 is expressed in the pneumocytes of the lungs and in medullary epithelial cells, including Hassall’s corpuscles of the thymus. Trop2 is highly expressed in the breast; lung adenomas; luminal structures; and the cervix, prostate, skin, and oesophagus (Dum et al., 2022). Importantly, Trop2 is overexpressed in many tumor types, including pancreatic cancer, breast cancer, ovarian cancer, prostate cancer, colorectal cancer, and gastric cancer as well as squamous cell carcinoma of the oral cavity (Lombardi et al., 2023; Shen et al., 2021; Zaman et al., 2019). In contrast, the protein expression of Trop2 in most adenoid cystic carcinoma and myoepithelial is not upregulated compared with that in normal tissues(Wolber et al., 2021). Therefore, Trop2 has emerged as a very promising and strategic target for the advancement of ADCs in therapeutic development.

Trodelvy (sacituzumab govitecan, SG) was the first Trop2-targeting ADC that was approved for commercial use. In April 2020, the FDA approved it for the treatment of adult patients with metastatic triple-negative breast cancer (mTNBC) who had received at least two prior therapies for metastatic disease. Common side effects include but are not limited to leukopenia (decrease in white blood cells), anaemia (decrease in red blood cells), nausea, rash, loss of appetite, and abdominal pain (Schlam et al., 2023). According to the 2017 safety data, among 408 patients with solid tumors who received at least one dose of sacubitril, approximately 1% experienced serious adverse reactions (SARs), such as febrile neutropenia and dyspnoea, as well as more severe conditions, such as pleural effusion, pneumonia, and dehydration. More than 5% of patients reported Grade 3–4 adverse reactions, specifically neutropenia (43%), anaemia (12%), diarrhoea and hypophosphataemia (9% each), fatigue and febrile neutropenia (8% each), vomiting (6%), and dehydration (5%). Notably, severe neutropenia and diarrhoea, which may be life-threatening, have been included in the black box warning (Wahby et al., 2021). The wide range of adverse reactions associated with Trodelvy use reflect the extensive expression of Trop2 in normal tissues. The complex expression profile of the TROP-2 target indicates substantial potential for the development of future drugs that target TROP-2. Exploring combination therapies that target other molecules, such as ErbB3, to increase the efficacy of TROP-2-targeting ADCs is crucial. Further investigation into the cellular signalling pathways in which TROP-2 participates could identify new therapeutic targets and strategies to increase efficacy while reducing toxicity (Goldenberg et al., 2018).

### EGFR

Epidermal growth factor receptor (EGFR) is a transmembrane tyrosine kinase receptor and one of the four members of the erbB family of tyrosine kinase receptors. The activation of EGFR promotes cellular proliferation and differentiation, and it plays crucial roles in embryonic development, maintenance and repair of adult tissues, and proliferation and metastasis of cancer cells (Liang et al., 2020; Subramaniyan et al., 2022). EGFR overexpression can activate downstream signalling pathways, resulting in uncontrolled cellular growth and increased tumor cell proliferation and metastasis, ultimately leading to tumorigenesis (Darmadi et al., 2024). EGFR is expressed in most human tissues, showing weak positivity primarily in the skin, liver, gastrointestinal tract, and cervix, although its expression levels vary (London and Gallo, 2020). EGFR expression is crucial for normal cellular and physiological functions. However, EGFR overexpression is observed in various cancers, including head and neck tumors, non-small cell lung cancer (NSCLC), breast cancer, colorectal cancer, ovarian cancer, prostate cancer, renal cancer and glioblastoma, with a notably higher positivity rate in patients with low differentiation or metastasis (Liu et al., 2020; Nair et al., 2022; Uribe et al., 2021). In gastric cancer, the EGFR protein is expressed across samples but with significant variability (Galizia et al., 2007). The crucial physiological functions of EGFR and the adverse reactions induced by targeting EGFR highlight the complexity of targeting this receptor in cancer treatment. Thus a thorough understanding of the impact of EGFR-targeting therapies on patients, particularly the potential adverse reactions that may arise in patients with potential EGFR overexpression, is needed.

In patients with solid tumors that potentially overexpress EGFR, AbbVie’s ABBV-221 caused adverse effects that were primarily associated with frequent infusions. Specifically, symptoms related to infusion, which were observed in more than one patient, included rash (including maculopapular rash; 28.9%), pruritus (including generalized itching; 24.4%), flushing (8.9%), chills (6.7%), urticaria (6.7%), hypotension (4.4%), and nausea (4.4%). Other adverse events that were induced by the treatment included neutropenia (4.4%) and eye disorders (11.1%), which included blurred vision (6.7%), dry eyes (4.4%), keratitis (2.2%), and visual impairment (2.2%) (Cleary et al., 2020). Moreover, another AbbVie product, depatuxizumab mafodotin, reached phase III clinical trials, but these trials were terminated because no significant difference was observed compared with the placebo and Grade 3–4 ocular toxicity occurred in 14% of the participants (Lassman et al., 2023). The termination of studies on AVID100 in 2021 due to a lack of efficacy may be attributed to a combination of factors, including inadequate drug targeting, severe adverse reactions, and tumor heterogeneity. These factors collectively impact the effectiveness and feasibility of AVID100. When developing therapeutic strategies that target EGFR, it is crucial to comprehensively consider these factors and continuously optimize treatment approaches to increase efficacy and minimize adverse reactions.

In September 2020, Akalux was approved in Japan for the treatment of unresectable, locally advanced, or recurrent head and neck cancer. In this ADC, the photosensitizer IRDye700DX is conjugated to an antibody, and near-infrared light is used for activation to induce tumor cell death (Gomes-da-Silva et al., 2020). Ongoing phase I/II clinical trials are exploring the use of Akalux for treating other cancers, including oesophageal and gastric cancer (Kobayashi and Choyke, 2021). To date, no significant adverse effects have been reported. Adverse effects associated with ADCs that target EGFR can be found in most normal tissues that express EGFR in the human body. Therefore, in the design of ADC drugs, the antibody component must be designed considering not only the affinity and specificity but also the distribution of the targeted antigen across various tissues in the body.

### Claudin-18

The Claudin-18 protein is a tetraspanin that is encoded by the multigene CLDN family and is a key component of tight junctions; it plays roles in the proliferation, differentiation, and migration of tumor cells (Cao et al., 2022). Claudin-18 serves as a highly selective biomarker, with limited expression in normal tissues; it is typically hidden within the supramolecular complexes of tight junctions and is expressed only on the surface of differentiated epithelial cells in the gastric mucosa (Singh et al., 2017). However, during malignancy, tight junction proteins can become disrupted, exposing Claudin-18 epitopes on tumor cells.

Abnormal expression of Claudin-18 is frequently observed in the development and progression of various primary malignant tumors, including gastric cancer, pancreatic cancer, oesophageal cancer, ovarian cancer and non-small cell lung cancer (Liu et al., 2022a). Claudin-18 is highly expressed at tumor sites within the human body, whereas it is expressed at low levels or not at all in normal tissues, making it an ideal therapeutic target. Adverse reactions have not been reported in clinical trials to date. According to the PharmSnap database, there are currently 18 ADC therapies that target Claudin-18 in development. These include one in phase II, two in phases I/II, six in phase I, eight in preclinical stages, and one in the drug discovery phase, with no ADCs yet on the market. CMG-901, which was developed by Connoa/Leap Therapeutics, was the world’s first Claudin-18-targeting ADC to enter clinical development, and the fastest-progressing Claudin-18-targeting ADC, RC-118, which was developed by RemeGen, has entered phase II clinical trials in China; the latter is mainly used for treating Claudin-18-positive solid tumors. To date, no serious adverse effects related to these ADCs have been identified, and the primary reason may be closely associated with the distribution of Claudin-18 in the human body. Compared with other targets, Claudin-18 is expressed at relatively low levels in normal tissues because of its tight junction structure, and it is typically expressed only on the surface of epithelial cells at varying degrees of differentiation within the gastric mucosa (Gina Battaglia, 2023; Xu et al., 2024). Conversely, Claudin-18 is highly expressed in malignant tumors, particularly in gastric cancer, making it a better target. Furthermore, the anti-Claudin-18 antibody demonstrates remarkable specificity, synergizing with the targeted approach of ADC therapy to provide robust support for effective treatment without eliciting severe adverse reactions. Moreover, the linker–payload remains relatively stable. Collectively, these factors contribute to the precise targeting of ADCs to tumor cells while minimizing the impact on other normal tissues. With ongoing clinical trials and further research, we will gradually reveal any adverse effects of CMG-901, RC-118, or other ADCs that target Claudin-18. Concurrent in-depth investigations into this target will also gradually increase our understanding of the distribution of Claudin-18 within the human body. This knowledge will facilitate the future development of new drugs that target Claudin-18 with the aim of mitigating potential adverse effects.

### TF

Tissue factor (TF) is a transmembrane glycoprotein that is regulated by numerous transcription factors that are sensitive to hypoxia or anoxia, and it is involved in numerous pathological processes, such as thrombosis, metastasis, tumor growth, and angiogenesis (Heidari et al., 2024; Unruh and Horbinski, 2020). Under normal physiological conditions, TF primarily resides on cell surfaces not exposed to circulating blood, including the external membranes of blood vessels, capsules around organs, skin, mucosal epithelia, alveolar macrophages, and dendritic cells in certain lymph follicles. The constitutive expression of TF can also be observed in the cortex of the brain, glomeruli, cardiomyocytes, mucosal epithelium of the large bronchi and related submucosal glands, glial and neuronal cells within the eye, and mature and newly formed blood vessels (Drake et al., 1989; Hassan et al., 2023). The cells that form the fibrous capsules of organs, such as the liver, spleen, kidneys, and adrenal glands, exhibit uniform positive TF expression, whereas the respiratory, gastrointestinal, and bladder mucosa exhibit positive but variable TF expression, and in the bladder, the transitional epithelium shows weak positivity (Belting et al., 2004; Drake et al., 1989; Rong et al., 2006). The distribution of TF in these tissues and organs is vital for maintaining blood coagulation homeostasis and facilitating haemostasis after injury. TF expression can also be transiently upregulated by cytokines or growth factors in monocytes, macrophages, and endothelial cells (ECs). TF is notably overexpressed in patients with various types of cancer, including breast cancer, lung cancer, prostate cancer, gastric cancer, hepatocellular carcinoma, retinoblastoma, bladder cancer, oesophageal cancer, pancreatic cancer, colorectal cancer, cervical cancer and head and neck cancer (Ahmadi et al., 2023; Rondon et al., 2019).

Tivdak (tisotumab vedotin) is a first-class ADC that targets TF. The FDA granted accelerated approval for Tivdak on September 20, 2021, making it the first and only approved treatment for adult patients with recurrent or metastatic cervical cancer that progresses during or after chemotherapy (Luu et al., 2023). Throughout the clinical period, dose-escalation studies have been conducted on various tumors, including bladder cancer, cervical cancer, endometrial cancer, oesophageal cancer, non-small cell lung cancer, ovarian cancer, prostate cancer, and head and neck cancer. Among 147 patients, the most commonly reported adverse events were epistaxis (69%), fatigue (56%), nausea (52%), hair loss (44%), conjunctivitis (43%), decreased appetite (36%), constipation (35%), diarrhoea (30%), vomiting (29%), peripheral neuropathy (22%), dry eye (22%), and abdominal pain (20%). Eighty-two patients (56%) experienced Grade 3 or higher treatment-related adverse events. The most common treatment-emergent Grade 3 or higher adverse events included fatigue (10%), anaemia (5%), abdominal pain (4%), hypokalaemia (4%), conjunctivitis (3%), hyponatremia (3%), and vomiting (3%). Additionally, 41% of patients experienced at least one Grade 3 or higher treatment-related adverse event associated with the study drug (de Bono et al., 2019).

These data indicate that adverse reactions to Tivdak predominantly occur in the respiratory, gastrointestinal, and ocular systems, which is consistent with the distribution of TF expression in normal tissues. This correlation suggests that the distribution of TF expression in normal tissues may lead to off-target effects associated with the medication.

### FRα

Folate receptor alpha (FRα), also known as folate receptor 1 (FOLR1), is a cell membrane folate-binding protein that is encoded by the FOLR1 gene. It has a high affinity for folate, which is crucial for DNA synthesis, repair, and methylation (Scaranti et al., 2020). The expression pattern of FRα in normal human tissues is specific, with the highest expression recorded in the placenta (Gilmore et al., 2022). FRα is detected in the kidneys, lung epithelial cells, and choroid plexus of the brain, as well as in the thymus and bone marrow, which are active sites of cell division and immune cell production (Radziejewska and Chmurzynska, 2019; Varaganti et al., 2023).

Additionally, research has shown that FRα is involved in regulating tumor cell proliferation and metastasis. High expression of FRα has been detected in ovarian cancer, breast cancer, endometrial cancer, cervical cancer, lung cancer, colon cancer and mesotheliomas (Dindere et al., 2022; Gonzalez et al., 2024; Norton et al., 2020; Saito et al., 2023; Shi et al., 2022; Varaganti et al., 2023). The expression level of FRα varies across different cancer types and individual patients, potentially correlating with specific cancer subtypes, stages, and even tumor aggressiveness. Moreover, the expression of FRα may change with tumor progression and the response to treatment.

Elahere, which was developed by the Zai Laboratory/ImmunoGen, became the first FRα-targeting ADC to reach the market. In November 2022, it received accelerated FDA approval as a monotherapy for treating adults with FRα-positive, platinum-resistant epithelial ovarian cancer, fallopian tube cancer, or primary peritoneal cancer who had previously undergone 1–3 systemic treatment regimens (Heo, 2023). Between June 2020 and May 2021, 105 patients were enrolled in the study (NCT04296890). The most common treatment-related adverse events (TRAEs) were haematotoxicity, peripheral neuropathy, and ocular toxicity. The prevalent haematotoxic events included neutropenia (Grade 3, 2%), thrombocytopenia (Grade 3, 2%), and anaemia (Grade 3, 1%). Nineteen patients (18%) experienced peripheral neuropathy of Grade 1 (13%) or Grade 2 (5%); no ≥Grade 3 events was reported. Ten patients (9%) reported Grade 1 peripheral neuropathy at the start of the study. Severe (≥Grade 3) TRAEs were reported in 9% of patients. TRAEs led to dose delays in 33% of patients and dose reductions in 20% of patients. Overall, 9% of patients discontinued treatment due to TRAEs; one patient discontinued treatment due to Grade 4 corneal disorder, and the other discontinued treatment due to TRAEs, including thrombocytopenia (Grade 1 and 3), fatigue, infusion-related reactions, and sensory neuropathy (all Grade 3) (Matulonis et al., 2022). The observed ocular toxicity may be caused by DM4, as similar toxicities have been observed in patients treated with other antibody‒DM4 conjugates (Hafeez et al., 2020). FRα has been reported to be negatively expressed in the eye, although some studies have reported its expression in the conjunctiva (Mai et al., 2023); thus, the exact cause of ocular toxicity is unclear (Mai et al., 2023). Elahere, which is a pioneering FRα-targeting ADC, has exhibited a favourable benefit-risk safety profile in the treatment of platinum-resistant epithelial ovarian cancer, fallopian tube cancer, and primary peritoneal cancer. Nonetheless, the emergence of adverse events, notably haematotoxicity, peripheral neuropathy, and ocular toxicity, warrants attention, with a specific emphasis on ocular toxicity. Further investigations are needed to delineate the mechanistic pathways underlying ocular toxicity. The market debut of Elahere presents patients with a novel therapeutic avenue, albeit necessitating vigilant monitoring and adept management of potential adverse events to maintain patient safety and ensure efficacious treatment outcomes.

### HGFR

The HGFR protein, which is also known as c-MET, serves as a tyrosine kinase receptor for hepatocyte growth factor (HGF) and plays a critical role in embryonic development, wound healing, and tissue regeneration. In normal adult tissues, the expression and activation of HGFR are strictly regulated, and HGFR is most highly expressed in the liver, gastrointestinal tract, endometrium, and ovarian epithelial cells, as well as in oesophageal and skin basal keratinocytes (Zhao et al., 2022). HGFR-specific mRNAs have also been identified in the lungs, kidneys, thyroid, pancreas, and placenta, although immunofluorescence analysis has indicated very low HGFR protein expression in these organs (Bahrami et al., 2017). The functionality of HGFR signalling depends on its proper localization on the cell surface. For example, when HGF binds to HGFR, it induces receptor dimerization and autophosphorylation, activating a cascade of downstream signalling pathways that promote cell proliferation, mobility, and morphogenesis (Wang et al., 2020b). Abnormal HGFR expression and signalling can lead to various conditions, including chronic inflammation and cancer. In cancers, HGFR is often overexpressed or its activation is dysregulated, driving tumor growth, invasion, and metastasis in cancers including but not limited to non-small cell lung cancer (NSCLC), gastric cancer, colorectal cancer, renal cell carcinoma (RCC), head and neck squamous cell carcinoma (HNSCC), triple-negative breast cancer, ovarian cancer, pancreatic cancer, and epithelial tissue tumors (Kim et al., 2017; Mori et al., 2021; Yang et al., 2022).

High expression of HGFR can be attributed to gene amplification, transcriptional upregulation, or activating mutations. In some cancers, elevated HGFR activity is associated with a poor prognosis because of its role in promoting angiogenesis, tumor cell invasiveness, and metastasis. Therefore, HGFR is a target for cancer therapies, and various HGFR inhibitors and ADCs are in development or clinical trials. AbbVie’s candidate drug ABBV-399 is currently the only HGFR-targeting ADC in phase III clinical trials worldwide and is the most advanced ADC project in the AbbVie pipeline.

In January 2022, ABBV-399 (also called Teliso-V) was granted the Breakthrough Therapy designation by the FDA for treating advanced or metastatic, platinum-treated, HGFR-overexpressing, EGFR wild-type nonsquamous NSCLC patients. The most common adverse events (AEs) among the 136 enrolled patients were peripheral sensory neuropathy (25.0%), nausea (22.1%), and hypoalbuminaemia (20.6%). Two patients experienced Grade 5 AEs potentially related to ABBV-399, with one case of sudden death and one case of pneumonia in the SQ cohort (Verma et al., 2023). On November 29, 2023, AbbVie announced the top-line results from the single-arm Phase II LUMINOSITY trial of ABBV-399 for treating HGFR-overexpressing, EGFR wild-type, advanced/metastatic nonsquamous NSCLC. The results revealed an overall response rate (ORR) of 53.8% in the high HGFR expression group and 25.0% in the moderate HGFR expression group. The median durations of response were 9 months and 7.2 months, respectively, with median overall survival times of 14.6 months and 14.2 months, respectively. The results, thus far, are promising.

### CEACAM5

CEACAM5, which is also commonly referred to as carcinoembryonic antigen (CEA), is a cell surface glycoprotein belonging to the carcinoembryonic antigen-related cell adhesion molecule (CEACAM) family (Ambrosi et al., 2020). CEACAM5 is broadly utilized as a tumor marker in clinical settings, particularly for colorectal cancer (Huang et al., 2024). In normal tissues, the expression of CEACAM5 is largely restricted and is primarily found in the kidneys, bladder, larynx, pharynx, skin, submandibular gland, colon, oesophagus, duodenum, salivary glands, and sublingual gland. CEACAM5 is predominantly localized within the columnar and goblet cells of the colon, especially in the upper third of the crypts and the luminal surface (Han et al., 2020). The distribution of CEACAM5 is tissue dependent. For example, within glandular cells, such as those in the duodenum, salivary glands, submandibular gland, colon, and sublingual gland, CEACAM5 is located in the apical and/or lateral membranes. It is also found in the stratified squamous epithelium of the oesophagus, larynx, epiglottis, and bladder. Additionally, CEACAM5 is expressed in the basal cell membranes of the skin and the endothelial cells of the glomerulus. In contrast, CEACAM5 expression is not detected in the lung tissue, while demonstrating low expression levels in normal gastric and other nonmalignant tissues (Decary et al., 2020). However, its expression is significantly increased in several adenocarcinomas, including colorectal, gastric, pancreatic, breast cancer, non-small cell lung cancer (NSCLC), medullary thyroid carcinoma, and ovarian cancer (DeLucia et al., 2021; Han et al., 2020; Xu et al., 2022; Zhou et al., 2015). It is well known for its role in gastrointestinal tumors, especially colorectal cancer, and serves as a biomarker for diagnosis and disease management. Moreover, owing to its differential expression in normal and malignant tissues, CEACAM5 is being explored as a target for immunotherapy and drug delivery in cancer treatment.

Tusamitamab ravtansine (also called SAR408701/IBI-126) is a novel ADC that targets CEACAM5 that was developed by Sanofi and introduced through a collaboration with ImmunoGen. It represents the forefront of research targeting CEACAM5 and has entered phase III clinical trials. This drug is composed of a humanized monoclonal antibody (SAR408377) conjugated to DM4 through a cleavable linker (SPDB) that was developed by ImmunoGen. Its utility was assessed in patients with high CEACAM5 expression who had previously undergone treatment for metastatic nonsquamous (NSQ) NSCLC. In a published open-label phase 1/2 study (NCT02187848), which included 92 heavily pretreated NSQ NSCLC patients with high (*n* = 64) or medium (*n* = 28) CEACAM5 expression, the most common TRAEs were corneal events (keratitis/corneal disorders). As of December 2021, among 11 patients who were treated for more than 12 months, 8 patients (73%) experienced corneal adverse events, and 4 patients (36%) were classified as Grade ≥ 3 (Ricordel et al., 2022). Recently, Sanofi announced the termination of the global clinical development program for tusamitamab ravtansine. This decision was primarily based on analysis of interim data from the phase III CARMEN-LC03 study, which evaluated the drug as a second-line treatment for CEACAM5-positive metastatic NSQ NSCLC. The results revealed that although there was a trend towards improved overall survival (OS) with tusamitamab ravtansine monotherapy compared with docetaxel monotherapy, it did not meet the dual primary endpoint of progression-free survival (PFS).

### Nectin-4

Nectin-4, which is also known as PVRL4, is a cell adhesion molecule that is involved in the formation of adherens junctions, and it plays roles in cell‒cell adhesion, proliferation, migration, and differentiation. It is part of the nectin family, and it is a crucial component of the cell adhesion complex (Duraivelan and Samanta, 2021; Liu et al., 2021). In normal tissues, nectin-4 is expressed mainly during embryonic development, suggesting its potential importance in placental development and function (Chatterjee et al., 2021). Nectin-4 has been found to be overexpressed in various human cancers, including triple-negative breast cancer, ovarian cancer, lung cancer, pancreatic cancer, gastric cancer, melanoma, cutaneous squamous cell carcinoma oesophageal cancer, bladder cancer, urothelial cancer and head and neck squamous cell carcinoma (Bouleftour et al., 2022; Deng et al., 2019; Hashimoto et al., 2022; Heath and Rosenberg, 2021; Tanaka et al., 2021; Zhang et al., 2018).

Nectin-4 has been identified as a potential biomarker of cancer progression and prognosis. Its role in cell adhesion, motility, and proliferation makes it a likely target for therapeutic intervention. For example, the ADC enfortumab vedotin, which targets Nectin-4, has been approved for the treatment of locally advanced or metastatic urothelial cancer that is unresponsive to other types of treatment, such as chemotherapy or immunotherapy. However, this ADC appears to exert toxic effects particularly in the cornea. Common side effects of enfortumab vedotin include dry eyes, keratitis, blurred vision, excessive tearing, and limbal stem cell deficiency (Rosenberg et al., 2019). Other treatment-related adverse events are fatigue (≥Grade 3.6%), decreased appetite (≥Grade 3.1%), peripheral sensory neuropathy (≥Grade 3.2%), hair loss (49% across all grades), and speech disorders (40% across all grades) (Sakellakis et al., 2022). The expression and distribution of Nectin-4 in normal tissues remain to be fully elucidated, and the severe ocular toxicity observed cannot yet be rationally explained. However, with more in-depth research on the Nectin-4 target, ADCs that target Nectin-4 could broaden our understanding of its role in cancer and its potential as therapeutic targets.

## Current challenges in the safety and toxicity of ADCs and response strategies

ADCs offer an innovative approach to cancer treatment, as they target tumors with high specificity while aiming to minimize the impact on healthy, normal cells. However, despite the conceptual promise and some clinical successes of ADCs, there are still considerable obstacles that have led to the failure of many candidate drugs in clinical trials. These challenges are often associated with safety and efficacy issues. After administration, ADCs gradually diffuse through the bloodstream into the interstitium of body tissues and eventually reach target tumor cells; however, only an estimated 0.1% of the injected dose of ADCs actually reaches the solid tumor (Nagayama et al., 2017). This low delivery efficiency indicates that the vast majority of the drug does not effectively act on tumor cells. In circulation, the instability of the linker–payload of the ADC can result in premature payload release, and nonspecific adsorption can promote the internalization of the ADC into normal tissue cells, causing off-target toxicity. Furthermore, as many target antigens are also expressed in normal tissues and organs, ADCs can similarly target and be internalized by normal cells, leading to on-target toxicity. While the use of noncleavable or more stable linkers can partially mitigate off-target toxicity, the issue of on-target toxicity remains more severe. Another significant obstacle in ADC therapy is the development of resistance by tumor cells. The potential mechanisms underlying the development of resistance are diverse and include altered internalization of ADCs, mutation of target antigens or downregulation of their expression, and increased activity of efflux pumps on the cell surface.

Current research highlights two main challenges in the development of ADCs: the ability to accurately identify targets that are highly overexpressed in cancer cells but minimally expressed in normal tissues and the ability to overcome drug resistance. Presently, target selection primarily depends on available data about antigen protein expression levels. However, a thorough understanding of antigen distribution in normal tissues remains essential and requires further investigation.

### Identifying and refining antigen expression and distribution data in solid tumors

**Figure 4.**
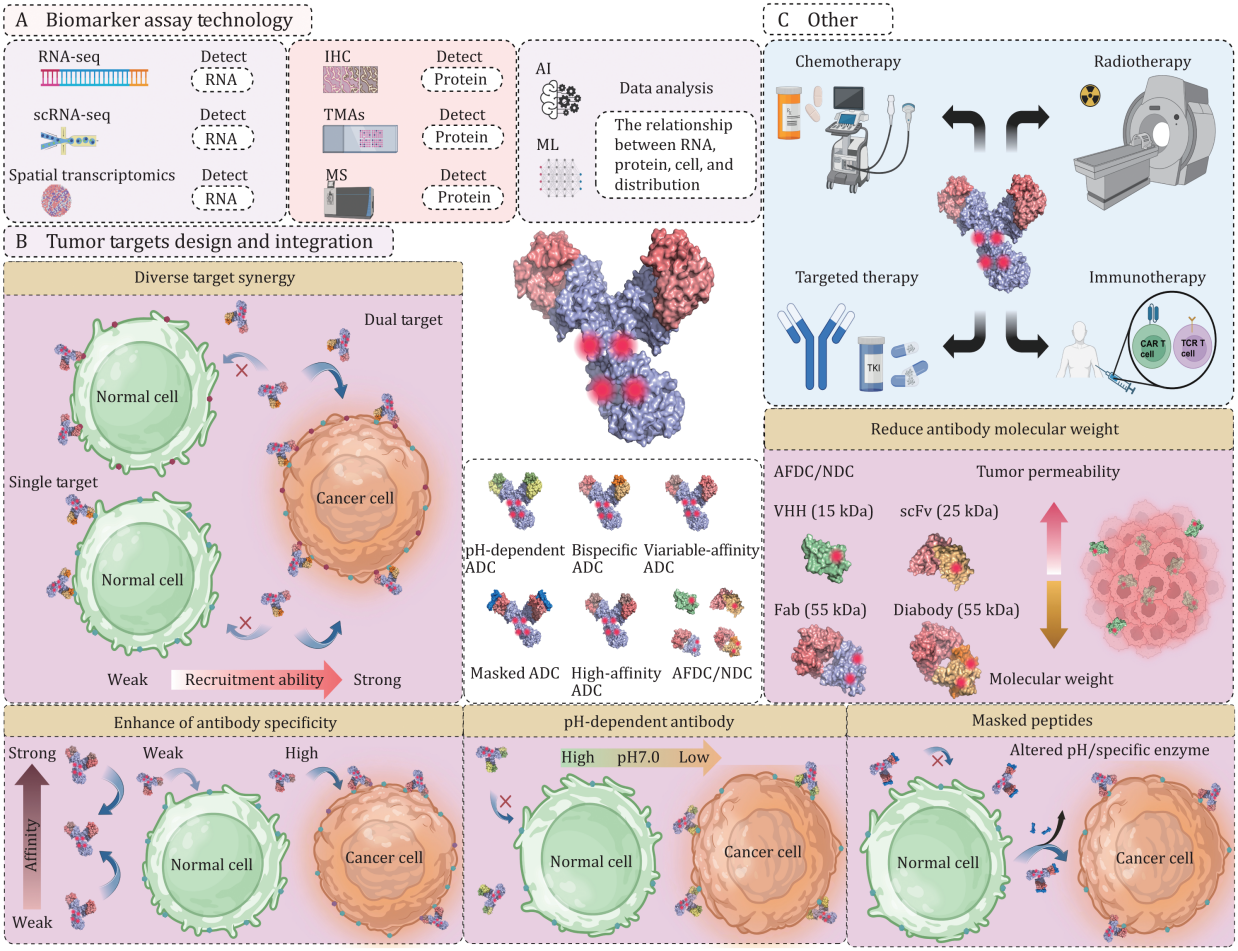
Strategies for ADC structural innovation and treatment optimization. (A) Systematic analysis of tumor target RNA and protein expression levels in normal and tumor tissues by improving data and using AI and ML as aids (top left). (B) Antibody engineering modifications, including redesign and combination of tumor targets (middle left, left side represents single-epitope ADC, right side represents dual-target/epitope ADC); high-affinity ADCs or ADCs with different affinity combinations (bottom left); pH-dependent ADCs (bottom centre, enabling no or weak binding to targets in neutral pH environments and strong binding in slightly acidic environments); AFDCs/NDC (bottom right, designing different structures such as VHH, scFv, etc.); and masked peptide ADCs (middle right, utilizing pH-responsive or specific enzyme cleavage for ADC release). (C) Combination therapies to improve efficacy and safety (top right, such as chemotherapy, radiotherapy, targeted therapy, or immunotherapy). Abbreviations: ADC, antibody-drug conjugate; AI, artificial intelligence; ML, machine learning; AFDC, antibody fragment drug conjugates; NDC, nanobody drug conjugate.

In the development of ADCs, mitigating side effects that are caused by the expression of target antigens in normal tissues is crucial. Several methods are demonstrated here for reference, as shown in Fig. 4. The initial step involves the comprehensive identification of tumor-specific antigens or antigens with minimal expression in normal tissues via high-throughput transcriptome sequencing (RNA-Seq) that involves gene expression analysis across various samples to identify potential targets. Subsequently, tissue microarrays (TMAs) and IHC are employed to quantitatively confirm the expression differences and localization of target proteins in an extensive array of tissue samples, ensuring their tumor-specific overexpression. Furthermore, mass spectrometry (MS) facilitates in-depth proteomic analysis, further confirming the expression levels and modification states of target proteins. Integrating these data with bioinformatics analyses enables more the precise evaluation of candidate antigen expression at specific tissue and cellular levels, ensuring that the selected targets exhibit high tumor specificity and thereby minimizing potential harm to normal tissue. Although these methodologies are relatively mature, the lack of systematic analysis has revealed significant discrepancies between gene and protein expression data for many targets, as revealed by database comparisons. The underlying reasons remain unclear, but one potential explanation is that overglycosylation of antigens might mask epitopes, resulting in weakly positive IHC results, and the use of different antibodies could lead to inconsistent detection results (Kern et al., 2023). Therefore, a systematic organization and comparison are imperative for making more accurate determinations, which will expedite the evaluation of drug developability and the prediction of potential safety issues in the future, thereby facilitating prompt strategic responses.

With advances in technology, single-cell sequencing (scRNA-seq) and spatial transcriptomics have emerged as pivotal tools for analysing the expression of antigens within heterogeneous tumors and complex microenvironments. scRNA-seq enables the identification of differential antigen expression patterns among various cellular subpopulations within tumors, whereas spatial transcriptomics allows the highly detailed and precise visualization of antigen distribution within tumors and adjacent normal tissues. Combined with IHC, these technologies can map the specific localization of antigens within tissues, aiding researchers in understanding the distribution of target antigens under both healthy and diseased states. This information is crucial for understanding the dynamic expression of antigens during tumor progression and their roles in the local microenvironment. Moreover, leveraging bioinformatics to integrate and analyse extensive data from public databases can enhance the systematic understanding of identified antigens, potentially leading to the discovery of novel ADC targets. This process is key to reducing nonspecific targeting and minimizing side effects. As cell- and molecular-level data become increasingly abundant, artificial intelligence (AI) and machine learning (ML) technologies are progressively becoming invaluable in the discovery and validation of antigens. With the use of sophisticated tools and methodologies to facilitate antigen identification, evaluate antigen‒antibody interactions, and assess off-target effects, these technologies optimize key facets of antigen research. AI and ML can be used to identify promising antigen candidates from vast bioinformatics databases, confirm their association with specific cancers through gene expression profile analyses, and predict the binding affinity and specificity between antibodies and antigens, thus streamlining the process of screening antigens and candidate antibodies. Moreover, these tools can facilitate the prediction of potential off-target effects, aid in the development of personalized treatment plans, and expedite the drug development process. Through meticulous and systematic analyses, researchers can more accurately predict the therapeutic potential of antigens, refine ADC designs, and provide guidance for clinical trial designs and patient selection, ensuring the safety and efficacy of ADC therapies.

### Rational design of drug targets and combinations to minimize off-target effects

The efficacy and safety of ADCs rely on a multifaceted approach that involves precise target identification, specific antibody engineering, cytotoxic drug (payload) selection, and linker chemistry. ADCs, which are as complex therapeutic agents, combine the high specificity of monoclonal antibodies with the potent efficacy of cytotoxic drugs, aiming to minimize systemic toxicity while delivering lethal doses to cancer cells. An analysis of market-approved and clinically failed ADCs revealed that single-target ADCs often cause off-target toxicity, which is a significant hurdle that is driven by the challenge of identifying targets that are exclusively expressed on tumor cells but not on normal tissues. Therefore, the rational design and combination of targets to circumvent off-target toxicity induced by single-target ADCs have emerged as pivotal strategies for expanding the therapeutic window of ADCs.

#### Multi-target selection

During the selection of targets for ADCs, priority is given to antigens that are overexpressed in tumors but minimally expressed in normal tissues. Identifying targets that fully meet these criteria can be challenging, thus leading to the consideration of combining two or more targets for ADC formulations. By analysing the mRNA transcription and protein expression levels of different targets in both tumor and normal tissues, those that are highly expressed across certain tumor types are chosen. Furthermore, through correlation analysis to identify intertarget relationships, highly correlated targets are combined, or different epitopes of the same antigen are targeted to increase the precision of the ADC and facilitate drug internalization. To mitigate the risk of off-target toxicity associated with the design of dual- or multitarget ADCs, in addition to ensuring the high tumor expression of the target proteins, the differentiated expression of target proteins in normal tissues is determined to avoid the use of antigens that are coexpressed in the same normal tissue. Additionally, leveraging genomics in functional genomics studies to identify antigens that are critical for target cell survival but nonessential for nontarget cells allows for the combination of key antigens with other preferred tumor targets, allowing the design of dual- or multitarget ADCs to minimize off-target side effects that are inherent in single-target approaches.

#### Increasing antibody specificity

To increase the affinity and specificity of antibodies, techniques such as phage or yeast display are utilized to optimize the variable regions of antibodies, improving their antigen-binding specificity and affinity. Additionally, site-directed mutagenesis that targets complementarity-determining regions can be performed to increase the specificity of antibody‒antigen interactions, reducing nonspecific binding to similar antigens that are presented on nontarget cells (Ye et al., 2022). Further refinements include adjusting antibody affinity on the basis of differences in antigen expression across cells and employing combinatorial strategies for in-depth screening to identify dual-target antibodies with optimal affinity combinations. Owing to their increased precision and specificity, these dual-target antibodies serve as ideal carriers for ADCs, facilitating the targeted treatment of tumors.

#### pH-dependent antibodies

A pH-dependent strategy that leverages the differential acidity between tumor microenvironments and normal tissues can be implemented to mitigate the off-target toxicity of ADCs. The acidic nature of the tumor environment (with pH values ranging from 6.5 to 7.0) is very different from the relatively neutral pH of normal tissues (with pH values ranging from 7.2 to 7.5) (Liu et al., 2022c; Wei and Sulea, 2024). This difference allows specially engineered antibodies to bind specifically to tumor antigens under acidic conditions, whereas their binding to normal tissues under neutral pH conditions is minimized or completely abrogated. The distinct advantage of this approach is its precise selectivity, which concentrates drug release within the acidic tumor milieu and significantly reduces the likelihood of the drug targeting normal tissue cells, thereby effectively minimizing harm to healthy cells.

Sensei Biotherapeutics is developing SNS-101, which is a conditionally activated monoclonal IgG1 antibody that has been tailored to selectively target the VISTA checkpoint under the acidic conditions of the tumor microenvironment. This checkpoint effectively suppresses T-cell activity via its interaction with the receptor PSGL-1 (Horst et al., 2022). Preclinical studies have demonstrated that SNS-101, when used as a monotherapy, has the capacity to inhibit tumor growth, significantly increase the antitumor efficacy of PD-1 inhibitors, and reduce the risk of cytokine release syndrome. The application of this engineered antibody technology to ADCs ensures that the active components of the ADCs selectively target tumor cells while reducing their binding to normal cells in the neutral pH environment of healthy tissues. This selective action minimizes the release of toxic agents in nontarget areas, substantially lowering the risk of off-target toxicity.

#### Masked peptides

Masking peptide technology is an innovative approach that can be used to address the challenge of the off-target toxicity of ADCs. Off-target toxicity, which results in nonspecific adverse effects due to a drug mistakenly attacking normal cells instead of only attacking tumor cells, has long been an obstacle in the development of ADCs. Masking peptide technology involves the integration of a peptide masking layer between the ADC’s antibody and its target-binding domain, effectively restricting its targeting to healthy tissues. Under specific conditions, such as altered pH or the presence of specific enzymes, these peptides are cleaved, while the drug remains in an inactive state until it reaches the target cells. This significantly reduces potential damage to normal cells, increasing the safety of therapeutic intervention.

Under this design paradigm, ADCs circulate through the bloodstream to the vicinity of tumor cells that express the target antigen. Upon encountering tumor-specific proteases, the masking peptide is specifically cleaved, revealing the antibody binding site, thus enabling the antibody to specifically recognize and bind to certain antigens on the surface of tumor cells. This leads to the internalization of the ADC by tumor cells, followed by its entry into the intracellular microenvironment, where lysosomal protease activity releases the carried toxin to exert cytotoxic effects on tumor cells. This precise control-release mechanism significantly mitigates adverse effects on normal tissues, profoundly enhancing the safety and efficacy of ADC therapies. Termed as Probody^®^ therapeutic technology, it has been used in various emerging immune therapies, including immune checkpoint inhibitors, bispecific antibodies, cytokine therapies and ADCs (Sanborn et al., 2021; Boustany et al., 2022). Within the field of ADC research, CX-2009 Probody-ADC, which targets CD166, and CX-2029 Probody-ADC, which targets CD71, have advanced to phase II clinical trials (Chomet et al., 2020). However, both studies were subsequently terminated owing to inadequate therapeutic indices, highlighting the persistent challenge of optimizing the delicate balance between safety and efficacy in ADC development.

#### Reducing antibody molecular weight

Conventional IgG-like ADCs are characterized by relatively large molecular weights, which results in limited tumor penetration and prolonged circulation times *in vivo*. These properties contribute to more pronounced side effects. Some researchers have aimed to reduce the molecular weight of antibodies, and have demonstrated that ADCs based on these low molecular weight antibodies exhibit enhanced tumor penetration compared to traditional IgG-like ADCs (Wu et al., 2022b; Zhu et al., 2024). Similarly, Huang et al. demonstrated that VHH-based drug conjugates can reduce drug accumulation in tissues and organs, providing a foundation for research aimed at minimizing the toxic side effects of ADC drugs (Huang et al., 2019). A pertinent example is MT-6420, an engineered toxin body with a molecular weight of 55 kDa (Barve et al., 2023). A clinical trial (NCT04795713) involving patients with metastatic nasopharyngeal carcinoma (NPC) and non-small cell lung cancer showed that MT-6420 was well-tolerated across five dose cohorts.

### Others

Combining ADCs with other therapeutic modalities is a widely adopted strategy to increase efficacy while maintaining safety. Such combinations may incorporate chemical agents, radiopharmaceuticals, targeted therapies, or immunotherapies. Combination approaches also have the potential to mitigate some of the adverse effects that are associated with ADC treatments.

Moreover, there are substantial opportunities for improving the stability and selectivity of linker–payload components in ADCs. Advances in these areas could significantly increase the targeting precision and overall stability of ADCs, thereby improving therapeutic outcomes and safety profiles. These refinements are critical for the continued development and success of ADC-based therapies in clinical settings. As the field progresses, such improvements will be instrumental in expanding the therapeutic window of ADCs and solidifying their position as a cornerstone of modern cancer treatment strategies.

## Conclusion

Cancer treatment strategies are diverse and include surgery, chemotherapy, radiotherapy, immunotherapy, and targeted therapy, among others. In this context, ADCs have emerged as novel therapeutic agents that integrate the precision of targeted therapy with the potency of chemotherapy, resulting in unique advantages. Compared with chemotherapy and radiotherapy, ADCs offer improved selectivity with reduced side effects; they possess greater target specificity than immunotherapy does and can mitigate resistance issues associated with certain targeted therapies. Nonetheless, the use of ADCs in cancer treatment faces challenges, including target selection, conjugate stability, and dose-limiting toxicity. Future research directions include a comprehensive analysis of target expression in various tumors and normal tissues, precise target selection, improved ADC design to address off-target concerns, increased drug accumulation at tumor sites, development of stable and nontoxic linker–payloads to address safety issues, expansion of therapeutic windows, and exploration of combination strategies with other therapies to increase cancer treatment efficacy.

The development of ADCs faces challenges, and precise studies on antigens that are associated with solid tumors are essential to advance ADCs from theoretical constructs to clinically useful tools. Accurate target identification and comprehensive expression analyses will increase the therapeutic efficacy of ADCs and significantly reduce potential toxicity to nontargeted tissues, offering safer and more effective treatment options for patients. Moreover, these endeavours highlight the critical roles of modern biotechnology and data science in cancer therapy, laying a robust foundation for personalized and precision medicine approaches in the future. Furthermore, these advances may broaden the application of ADCs beyond oncology to autoimmune diseases, inflammatory conditions, intractable infections, and arteriosclerosis, thus benefiting a wider patient population.
